# Nutrition interventions for healthy ageing across the lifespan: a conference report

**DOI:** 10.1007/s00394-019-02027-z

**Published:** 2019-06-28

**Authors:** A. Kalache, A. I. de Hoogh, S. E. Howlett, B. Kennedy, M. Eggersdorfer, D. S. Marsman, A. Shao, J. C. Griffiths

**Affiliations:** 1 International Longevity Centre Global Alliance, Rio de Janeiro, Brazil; 2 TNO, Zeist, The Netherlands; 3 Dalhousie University, Halifax, NS Canada; 4 National University Singapore, Singapore, Singapore; 5 University of Groningen, Groningen, The Netherlands; 6 Procter & Gamble Health Care, Cincinnati, OH USA; 7 Amway/Nutrilite, Buena Park, CA USA; 8 Council for Responsible Nutrition-International, Washington, DC USA

**Keywords:** Ageing, Bioactives, Biomarkers, Functional ability, Healthspan, Inequalities, Intrinsic capacity, Lifespan, Nutrition, Vitamins

## Abstract

Thanks to advances in modern medicine over the past century, the world’s population has experienced a marked increase in longevity. However, disparities exist that lead to groups with both shorter lifespan and significantly diminished health, especially in the aged. Unequal access to proper nutrition, healthcare services, and information to make informed health and nutrition decisions all contribute to these concerns. This in turn has hastened the ageing process in some and adversely affected others’ ability to age healthfully. Many in developing as well as developed societies are plagued with the dichotomy of simultaneous calorie excess and nutrient inadequacy. This has resulted in mental and physical deterioration, increased non-communicable disease rates, lost productivity and quality of life, and increased medical costs. While adequate nutrition is fundamental to good health, it remains unclear what impact various dietary interventions may have on improving healthspan and quality of life with age. With a rapidly ageing global population, there is an urgent need for innovative approaches to health promotion as individual’s age. Successful research, education, and interventions should include the development of both qualitative and quantitative biomarkers and other tools which can measure improvements in physiological integrity throughout life. Data-driven health policy shifts should be aimed at reducing the socio-economic inequalities that lead to premature ageing. A framework for progress has been proposed and published by the World Health Organization in its Global Strategy and Action Plan on Ageing and Health. This symposium focused on the impact of nutrition on this framework, stressing the need to better understand an individual’s balance of intrinsic capacity and functional abilities at various life stages, and the impact this balance has on their mental and physical health in the environments they inhabit.

## Introduction

The World Health Organization’s (WHO) definition of Healthy Ageing—*the process of developing and maintaining the functional ability that enables wellbeing in older age* [70]—continues to be a driver of both scientific research and policy advancement on ageing. The foundation of this definition is the functional ability of the individual, which in turn is dependent upon his/her intrinsic capacity and how that interacts with the environment (recently discussed in detail by Marsman et al. 2018) [41]. With a rapidly expanding global population over the age of 65 driven in large part by increased life expectancy, interventions and policies are needed to improve the intrinsic capacity and functional ability trajectories.

The Longevity Revolution (LR) is a term used to characterise the recent and rapid increase in life expectancy (or lifespan) overall which has not been paralleled by the same increase in healthspan (defined as *the period of life spent in good health, free from the chronic diseases and disabilities of ageing*) [25, 65]. Thus, although life expectancy has increased worldwide, the intrinsic capacity and therefore functional ability trajectories have not improved simultaneously. This is especially apparent in developing countries where social inequalities have led to premature ageing, characterised by reduced healthspan and increased rates of chronic disease and disability. To reduce premature ageing and promote healthy ageing, appropriate policies and interventions are needed both at the individual and societal levels.

The impact of diet and lifestyle on health status is now well established. In developing countries, while undernutrition (nutritional inadequacy) continues to be a public health challenge, it is now accompanied by over-nutrition—societies are overfed, yet undernourished, leading to the dichotomy of obesity in the face of nutrient inadequacy and premature ageing [55]. To foster research that will further the understanding of which nutritional interventions improve healthspan and by what mechanism, it is critical to identify biomarkers of ageing and healthy ageing that can be used to assess the impact of interventions.

Ageing research has evolved from relying on single, static biomarkers to a 360° systems-based approach [55, 64, 74], which incorporates biomarkers from a range of biological, psychological, functional, and even digital platforms (see Table 1).

**Table 1:** Biomarkers of healthy ageing

Biomarker	Description	Category
Healthspace	Multiple biomarkers that are combined into one or more composite scores (axes) using multivariate statistical methods to measure and visualize the body’s biologic response to an intervention (or stressor); serves as a comprehensive assessment of an individual’s health status	Biological
Frailty Index	Measurement of the accumulation of a collection of functional health deficits relative to the total number of possible deficits	Functional
Facial imageing	Measurement of physical changes in facial structure over time	Digital

Healthspace is an example of systems biology that can be used to assess and visualize the body’s biologic and physiologic response to a stressor or intervention on self-selected ‘axes’. The Frailty Index (FI) combines a series of functional deficits as an overall indicator of intrinsic capacity. Technological advances have afforded the use of digital imageing as a means to estimate ageing by analyzing physical facial traits. These measurements collectively assess the body’s ability to cope mentally and physically with the stress of the ageing process. Using a systems-based approach and combining these into indices may provide a more accurate overall indication of healthy ageing, and can more readily assess the impact of different nutritional interventions which in turn have the potential to be personalised.

Today, well-studied diet and lifestyle interventions shown to increase both lifespan and healthspan are limited. Caloric restriction, intermittent fasting and exercise each have demonstrated longevity-enhancing effects which are highly conserved among animal models and humans [58]. Few other nutrition interventions have been shown to have these effects. In contrast, several drug compounds have been shown to exert effects on lifespan that mimic the effects of caloric restriction and exercise. Rapamycin, Metformin and certain non-steroidal anti-inflammatory drugs (NSAIDS) are known to extend both lifespan and healthspan in animal models by inhibition of the mammalian target of rapamycin (mTOR) pathway [31]. That the mechanism by which caloric restriction and exercise increase lifespan and healthspan is also highly conserved and also works through the mTOR pathway, suggests that there may be promise for example, with certain dietary components that may also affect similar pathways. Resveratrol [53] and alpha-ketoglutarate [12] are among several promising naturally-occurring compounds that may enhance lifespan and healthspan via a similar mechanism.

It is well established that nutrient inadequacy is associated with increased risk for a variety of chronic diseases, especially when combined with obesity. Therefore, maintaining adequate nutrition status is important to reduce the risk of chronic diseases, many of which are age-related. However, whether achieving optimal status may also help delay premature ageing and support healthy ageing remains to be investigated.

Going forward, there is a need to establish widespread scientific agreement around which biomarkers are most indicative of healthy ageing and for additional research examining nutrition interventions that can positively affect these biomarkers. Research must also be aimed at the impact of early nutrition interventions, well before the onset of morbidity, as an approach to delay premature ageing. From a policy perspective, there is an urgent need to address social inequalities that contribute to premature ageing; interventions targeted at inequality are needed both at the individual and societal level. There is also an opportunity to assess the impact of nutrition interventions on age-related issues beyond health, such as healthcare cost and productivity.

## New paradigms in health assessment: 360° diagnosis, phenotypic flexibility and composite biomarkers

It has been widely accepted that healthy living can increase longevity. However, health care systems still focus on disease and mostly reductionist, pharmacological treatments [66]. This approach is inefficient for treating so-called “lifestyle-related diseases”, including metabolic syndrome, obesity, type-2 diabetes, and cardiovascular disease. Recent findings demonstrate that type-2 diabetes can even be reversed with long-term structural lifestyle changes [32, 62]. Also, lifestyle interventions have been proven successful in reducing obesity, metabolic syndrome and cardiovascular disease risk [10, 34, 48]. Adopting lifestyle-related interventions in healthcare requires a switch from professional-driven care to citizen–patient-centred approaches.

**Fig. 1:**
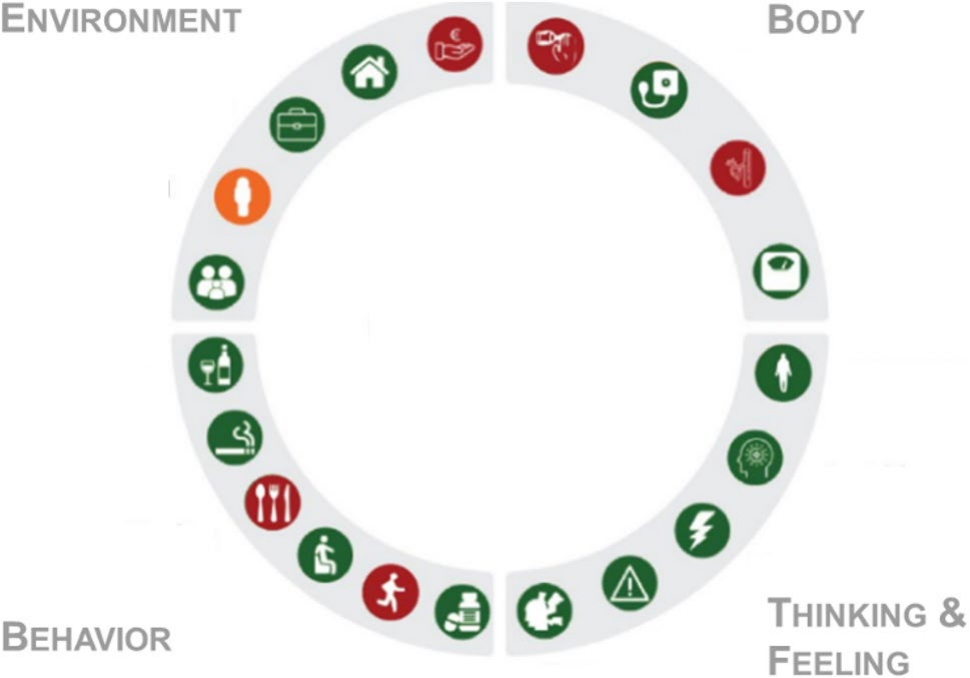
Visualization of the “profile wheel” resulting from an extensive 360° diagnosis. The profile wheel is constructed of four quadrants: environment, body, behavior and thinking and feeling. The quadrants are further split up in sub-domains, including glucose metabolism, body composition (body), medication use (behavior) and loneliness (environment). The colors represent the traffic light model, with green representing a healthy score for a sub-domain, orange an in-between state and red an unhealthy score

Previously in 2011, Huber et al. proposed to shift the focus in defining health from absence of disease towards “the ability to adapt and self-manage in the face of social, physical and emotional challenges” [20]. This definition advocates for more attention for the individual and their environment, instead of only focusing on standard clinical parameters. In other words, health should be approached as a system, taking into account the complex interplay between genetics, metabolic processes, lifestyle, psychological health and the socio-economic environment [64]. Using such a systems approach is not only of importance in health research, but also in health care practice. Research has shown that factors like motivation, comorbidities, mental health (e.g., depression), personality traits (e.g., self-efficacy) and the financial situation can negatively influence health behavior change and self-management [3, 46]. Such factors should thus be taken into account in developing an individual treatment plan. A so-called 360° diagnosis, ranging from personality questionnaires to sensors to sample analysis, can facilitate an extensive evaluation of the physical and mental health status of a patient, as well as their behavior and environment. The results can be visualized in a ‘profile wheel’, and as such offer a snapshot overview of a patient and their most pressing issues, and serve as a shared decision-making tool between patient and health care provider [64] (see Fig. 1).

The next challenge is to translate the results of such a 360° diagnosis into an effective treatment plan. Regular exercise, healthy eating, sufficient sleep and limiting or quitting unhealthy activities such as sedentary behavior, alcohol consumption and smoking, can contribute to increased lifespan and healthspan [13, 18]. However, it has recently been shown that individuals may respond differently to specific lifestyle interventions. For instance, type-2 diabetics seem to respond differently to dietary patterns, i.e., people with insulin resistance mainly in the liver respond better to a low-fat diet, whereas those with insulin resistance mainly in the muscles respond better to a Mediterranean diet [9]. It thus seems that personalization of lifestyle recommendations to the individual is necessary. Personalization of lifestyle advice on health status requires understanding of the compromised underlying metabolic processes for an individual and their ability to adapt to environmental challenges, also called ‘phenotypic flexibility’ [63]. A mixed-meal challenge test has been developed that is able to quantify phenotypic flexibility by measuring the responses in the pancreas, gut, adipose tissue, kidney, vasculature, muscle, liver, and metabolism as a whole using 132 different biomarkers [67]. The question is then, how to use the results of such a challenge test to determine systems flexibility?

**Fig. 2:**
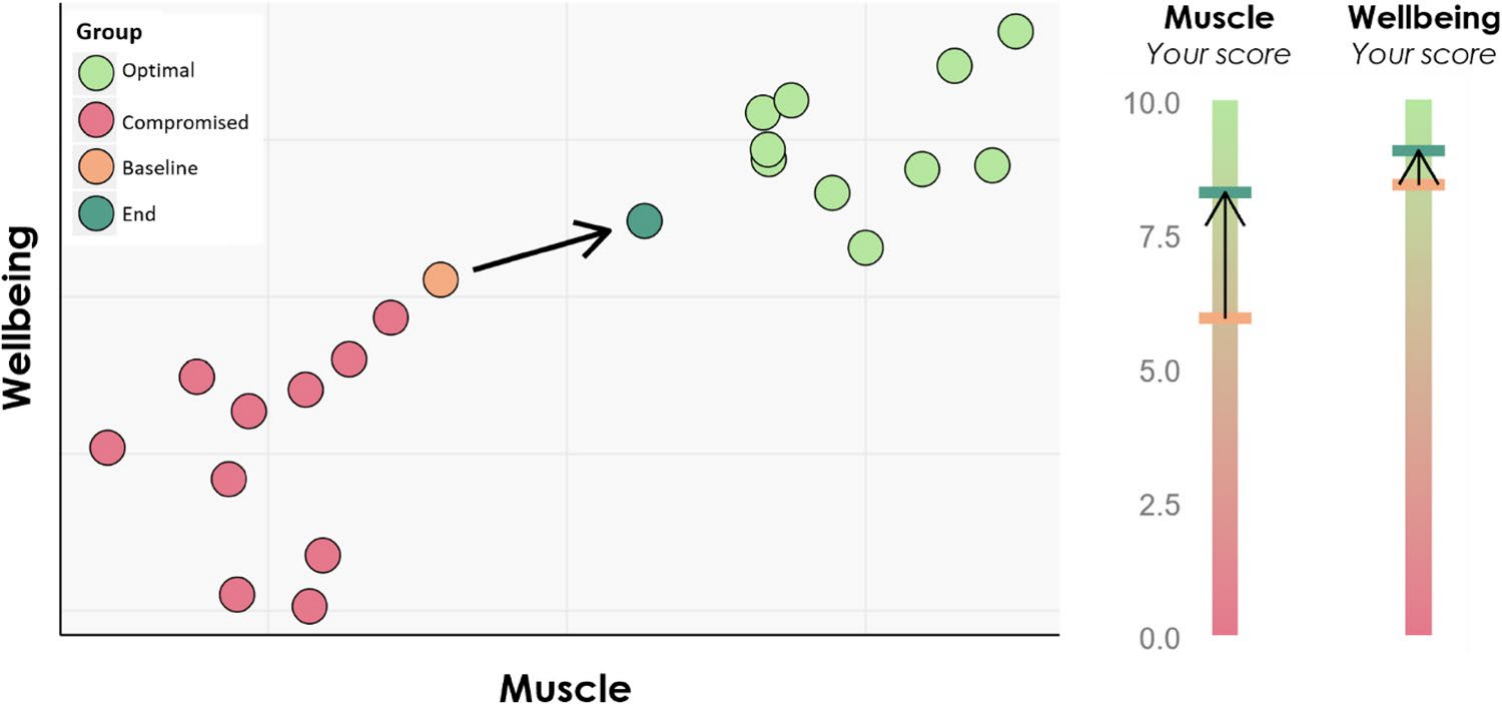
Example of a health space, showing the muscle health and wellbeing of an elderly person as compared to a reference group with optimal health and a reference group with compromised health status (no citation, paper under review)

Traditionally, single-parameter methods are used to measure aspects of flexibility, such as the oral glucose tolerance test. Such methods are only applicable if sufficient data from a range of health, disease and age conditions is available, which allows for establishing a desired health outcome as a benchmark for the measured outcome [61]. This, however, is again a reductionist approach, disregarding the complexity of systems health. A new concept called the “health space” can be used to visualize optimal systems flexibility, using a 2-days, 3-days or even 4-days space with predefined axes representing relevant biological processes or health areas of interest. These axes each consist of multiple biomarkers that are combined into a single score using multivariate statistical methods. Visualizations of a health space also provide a valuable tool in communicating health status (as compared to peers) to individuals, and to show changes in health over time (see Fig. 2).

To conclude, health research and health care should shift from reductionist disease management, to integral and personal treatment plans, including lifestyle. Various methods are being developed that enable a better understanding and quantification of health, using extensive 360° diagnosis tools, phenotypic flexibility for unraveling the underlying mechanisms and personalized treatment, and composite biomarkers as a measure of and benchmark for changes in systems flexibility. When combined, these methods can contribute to integrated, and more personalised health optimization.

### Biomarkers of frailty as a measure of healthspan in naturally-ageing mice

It is clear that people age at different rates, so that an individual’s biological age can be quite different from their calendar age. In other words, ageing is heterogeneous [44]. This has given rise to the concept of “frailty”, which is a state of increased vulnerability to adverse health outcomes [52]. Frailty is a key health care challenge because frail individuals are much more likely to experience poor quality of life, hospitalisation and death than their non-frail counterparts. Still, little is known about the biology of frailty, even though this could help understand critical determinants of healthspan and facilitate the development of novel interventions to treat frailty [52].

Recent research has pioneered the idea that frailty occurs not only in humans, but also in animal models of ageing (reviewed by Banga et al. [4]). Further, it has been shown that frailty can be quantified with a “frailty index” (FI) tool based on the accumulation of age-dependent health deficits; this technique is well established clinically and was originally developed for use in people [45]. To construct an FI, one can measure more than 30 readily observable signs of clinical deterioration in ageing mice. The number of deficits in an individual mouse, divided by the total number of deficits measured, yields an FI score that is theoretically between 0 (no deficits) and 1 (all possible deficits). Key features of FI scores are similar in mice and people [51]. For example, FI scores increase with age in mice with much the same trajectory as in people. High frailty scores also predict mortality in mice as in humans, the rates of deficit accumulation are virtually identical in mice and humans and the highest FI scores in mice (FI ≈ 0.55) approach the submaximal limit to frailty reported in humans [51]. In addition, female mice have higher frailty scores than males [27]. This is similar to the morbidity–mortality paradox seen in humans, where women have higher FI scores than men at any age, but paradoxically they live longer [17].

This powerful new FI tool has been used in a variety of preclinical studies. Research has shown that age-dependent maladaptive changes in heart function are correlated with, and closely graded by FI score [15]. The FI score also is responsive to interventions, as mice treated with known longevity interventions (e.g., caloric restriction, resveratrol) have lower scores than untreated animals [26]. Other mouse work also shows that chronic treatment with the angiotensin-converting enzyme (ACE) inhibitor, enalapril, attenuates frailty through sex-specific effects on pro- and anti-inflammatory cytokines [28]. Importantly, some of these findings in the mouse model have been translated back to humans. For instance, original work in mice showed that an FI could be developed based on routine laboratory blood work plus standard clinical test results (e.g., blood pressure, heart rate) [49]. Based on this, similar measures were used to develop a laboratory-based FI tool (FI-Lab) for use in people which predicts mortality just like the clinical FI tool [19].

As the FI score can be objectively quantified and shown to reflect normal physiologic/pathologic processes or responses to therapeutic interventions, this tool appears to meet the definition of a biomarker [7]. Therefore, this suggests that both the clinical FI tool and the laboratory-based FI tool may be useful as biomarkers of frailty and healthspan in ageing mice. This ability to quantify frailty in animals is a major step forward in the effort to understand the biology of frailty, and provides a new platform to develop and test novel clinical interventions.

## Impact of nutrition interventions on age-related decline: animal models and humans

We are at an interesting juncture, where research into the biology of ageing seems to have defined the major determinants of ageing and, more importantly, identified a robust and increasing number of interventions that have the potential to extend not just human lifespan but also healthspan, the disease-free and functional period of life [29, 35].

Interventions that may slow ageing include drugs (e.g., rapamycin, metformin) [5, 30], supplements (e.g., nicotinamide riboside, nicotinamide mononucleotide) [72], lifestyle interventions (e.g., exercise) [21] and diets (e.g., fasting) [42]. All of these and many others are reported to extend lifespan and/or healthspan in animal models. Moreover, many of these interventions have been explored in humans to varying degrees and in different, mostly disease-related contexts. While individual scientists certainly have their favorites, there is no consensus as to which might confer larger benefits and a nagging sense that ageing will ultimately be personal, such that successful interventions may be different from one individual to the next. This raises the question: how can we determine which interventions will (1) alter the rate of human ageing, (2) keep people healthy and functional longer and/or (3) prevent the onset of many age-related diseases simultaneously.

Determining whether interventions extend human life-span is a time- and cost-prohibitive strategy. In lieu of that, there are variations of two main themes. First, it is feasible to determine whether an intervention might protect against the onset of (or even reverse) an age-related disease or functional parameter linked to disease, and while often not favorable to the pharmaceutical industry because prevention trials are long and costly, this strategy has been tested. Perhaps most interesting are a pair of recent studies showing that mTOR inhibition by everolimus, alone or in combination with a mTOR catalytic inhibitor at low dose, can reverse aspects of immune senescence and protect against infections in individuals over 65 years of age [38, 39]. More comprehensively, it is possible to test whether an intervention protects against multiple chronic conditions of ageing simultaneously and this has been proposed with the TAME trial, which will test the effects of metformin in older individuals (notably, it will also look at biomarkers of ageing as secondary endpoints as follows) [47]. While appealing, this trial will involve thousands of individuals and have high cost, likely precluding a widespread comparison of different interventions at least in the near term.

A second approach involves determining whether interventions may alter the rate of change of ageing biomarkers and while interventions have gotten much attention, recent approaches to identify minimally invasive ageing biomarkers are equally important. While biomarkers of a specific disease have long been a major focus of research, ageing remained refractory to such indicators until recently. Through analysis of deep datasets usually in large populations and artificial intelligence-based approaches [14, 73], several biomarkers have been proposed, including the epigenetic clock [16], which measures the methylation status of specific DNA regions in the genome, and others including molecular markers, analysis of facial patterns, and even locomotor activity patterns [11, 50]. A potential advantage to trials using biomarkers is that they, at least in theory, (1) can be applied across a wider range of ages, (2) may have measurable effects in a shorter time window and (3) could be more economical, allowing for multi-intervention testing. However, it is still in the early days and much remains speculative. For instance, it is not clear whether (1) biomarkers are dynamic (although initial indications suggest this to be true), (2) biomarkers can be reversed or only changed in trajectory, and how (3) biomarkers relate to each other and disease onset. At this point, this approach is highly promising but also in its infancy.

The entry of ageing interventions into the human realm is exciting and potentially transformative, yet it remains underappreciated by key constituencies in the healthcare space. Government funding agencies have yet to fully embrace the concept, although the National Institutes of Health (NIH) National Institute of Ageing led Geroscience Initiative is encouraging [56]. Physicians, and even many geriatricians, remain unaware of the promise of interventions to extend healthspan, and others are skeptical that preventative medicine can succeed. Finally, regulatory agencies have yet to ponder whether ageing is a disease or condition and thus at present there exists no clear mechanism to approve ageing interventions, although the US Food and Drug Administration has recently displayed an open-minded approach. It is easy to look at these hurdles as inhibitory toward progress, but another exciting development has emerged. Recently, many investors have embraced the notion that human ageing can be delayed and have been convinced of the potential widespread quality-of-life and economic benefits that would accrue. Hence, private sector money has started to flow into the ageing space and now dozens of companies have emerged [56]. While no one knows the right path to success, the range of innovative ideas and approaches being tried dramatically increase the odds. Moreover, the imagination of the public is starting to be captured and it is incredulous to think that successful validation of interventions to slow ageing will not result into widespread dissemination. Once a backwater in medical research, the science of human ageing is now in the forefront and interventions extending human healthspan may be the biggest medical breakthroughs of this century.

## Supplementation’s role in assuring nutritional adequacy as one ages

One of mankind’s most remarkable achievements is increased life expectancy. Lifespan increased and continues to increase. Unfortunately, for many people this gain in life years is not matched by gain in years of healthy life. We face the challenge of a continuous increase of non-communicable diseases (NCDs) like osteoporosis, diabetes, cardiovascular diseases and cancer, to mention a few. There is growing evidence that lifestyle factors have substantial effects on health and wellbeing. The risk for non-communicable diseases can be reduced by lifestyle changes, with improved nutrition being an essential part. According to the World Health Organization (WHO) approximately one-third of cancers and up to 80% of heart disease, stroke and type-2 diabetes deaths are preventable [69]. To achieve this, a shift of focus from disease to capacity is required. Instead of diagnosing diseases on a point in time, trajectories across the life course should be monitored [41]. In this context the role and contribution of nutrients for a healthy life by achieving optimal status are of relevance [59]. Inadequate micronutrient intake and status are recognized to be an important factor in this context and an issue for millions. New findings and approaches which are used to assess the impact and benefit of nutrition providing an optimal nutrient status for a healthy life and healthy ageing six examples are further discussed:

- Improving mobility by counteracting muscle and bone loss.
- Supporting eye health via maintaining macular optical pigment density.
- Maintaining heart and vascular health via blood pressure and triglyceride management.
- Blood glucose management.
- Counteracting cognitive decline.
- Strengthening the immune system to reduce risk for respiratory tract infections.

Muscle and bone loss is a major issue in ageing. According to the International Osteoporosis Foundation (IOF) one in three women and one in five men over the age of 50 years will sustain an osteoporotic fracture. In women, the incidence of fractures is higher than the total incidence of cancer, heart infarction, stroke or diabetes. Osteoporotic fractures account for more days spent in hospitals than many other diseases, including diabetes, myocardial infarction, and breast cancer [23]. In several human studies, it is demonstrated that bone mass density increases with higher 25-hydroxyvitamin D [25(OH)D] plasma levels which can be achieved via different routes [8]. In an assessment of health care costs it was shown that the use of vitamin D food supplements by all Europeans age 55 and older diagnosed with osteoporosis or osteopenia then €3.96 billion in avoidable hospitalization costs per year are potentially realizable [54].

Millions of people are impacted globally by the deterioration of eye health. Macular degeneration is the leading cause of severe vision loss in people over age 50. Although macular degeneration is almost never a totally blinding condition, it can be a source of significant visual disability. Age-related macular eye disease (AMD) is ranked third by WHO as a priority eye disease, following closely behind cataracts and glaucoma, and AMD is the primary cause of blindness in industrialized countries. Macular Pigment Optical Density (MPOD) is a biomarker for eye health. In the Age-Related Eye Disease Study II (AREDS II) it was demonstrated that lutein and zeaxanthin supplementation reduced the risk of progression of Advanced AMD (AAMD) [2]. When people taking lutein and zeaxanthin (L/Z) alone or in combination with omega-3 fatty acids, i.e., DHA/EPA, were compared with people not taking L/Z they observed a 10% reduction of progression to AAMD in favour of L/Z. Additionally important when the analysis was conducted based on the level of L/Z dietary intake at baseline, the study demonstrated a 26% reduction in the risk of progression to AAMD for L/Z in persons with the lowest dietary intake of L/Z. A similar beneficial effect was also observed for cataracts. A recent assessment for the EU showed that a total potential of €6.20 billion in avoidable medical costs per year could be realized if all AMD patients used lutein and zeaxanthin supplement [54].

Cognitive impairment is a growing issue in the elderly. As people age (over 60 years) the brain starts to shrink at a rate of ~ 0.5%/year. Those with memory problems—‘mild cognitive impairment’—show a faster rate of shrinkage of ~ 1.0%/year and in patients with Alzheimer’s disease, the rate is higher still, at ~ 3%/year. B vitamin and omega-3 fatty acid status is inversely associated with the rate of shrinking of the brain and memory decline [24]. Undoubtedly nutrition has an important role in preserving cognitive and mental health and thus improving quality of life in older age. The impact of specific nutritional factors on brain health in ageing is an area of active investigation worldwide. Emerging evidence implicates subclinical deficiencies of certain nutrients in cognitive decline and poor mental health in older adults; however, the threshold for nutrient levels to prevent or delay declining brain health is still unknown. If the findings of ongoing studies described—which show promise in relation to B-vitamins, omega-3 fatty acids and polyphenols—are confirmed, a public health strategy to improve status of these key nutrients may help to achieve better cognitive and mental health in ageing. Future studies incorporating imageing techniques offer a robust basis for confirming effective nutrition interventions that could reduce the risk of cognitive and mental decline in ageing and the related burden on health services.

Diabetes is a fast-growing issue globally and is a heavy burden for health care systems [22]. Diabetes is the number 4 risk factor for mortality. Micronutrients play a role in the development and the progression of diabetes. Due to the high prevalence of diabetes, a nutritional approach may be an important contribution to society. The “vitamin D and Type-2 diabetes” (D2d) study is a multi-centred, randomized clinical trial in the US with the hypothesis that vitamin D supplementation may boost the body’s insulin production and processing. Study participants receive either vitamin D (4000 IU) supplement or placebo. The study is now complete, and currently data analysis is ongoing, with first results expected sometime in 2019 [60]. A nutritional solution could have a large impact on the quality of life for millions of people who are at risk for diabetes.

Health data from more than 190 countries show heart disease remains the number 1 global cause of death with 17.3 million deaths each year [68]. Direct and indirect costs of cardiovascular diseases (CVD) and stroke total more than $320 billion. Nutrition and lifestyle interventions have been identified as preventive factors. In the *VIT*amin D and Omeg*A*-3 Tria*L* (VITAL) in the US, vitamin D and omega-3 fatty acids were assessed for the prevention of cancer and CVD. Statistically significant reductions in secondary heart endpoints, including total and fatal myocardial infarctions (MI) and total coronary heart disease (CHD) in the omega-3 arm (total MI had a 28% risk reduction, total CHD had a 17% risk reduction, fatal MI had a 50% risk reduction) were achieved. The greatest reductions were observed in African Americans and those who do not eat a lot of fish. These results suggest that improving omega-3 status strengthens intrinsic capacity and provides a health benefit in primary prevention [40].

The loss of intrinsic capacity is associated with dysregulated immune and inflammatory responses. In a human study it was demonstrated that increasing vitamin E status reduces the risk for infections in the upper respiratory tract in elderly [43]. The group with the higher dose of vitamin E had lower infections rates (all respiratory infections had a 35% risk reduction, upper respiratory tract infections had a 38% risk reduction, common cold had a 37% risk reduction).

In summary it can be stated that the strengthening of the intrinsic capacity is relevant for a healthy life and ageing. A healthy lifestyle with a balanced diet, including food fortification and use of supplements providing all nutrients is one way to strengthen intrinsic capacity resulting in improved quality of life and healthy ageing. Monitoring trajectories across the life course is the recommended approach instead of waiting for the onset of disease(s). Intake recommendations for micronutrients (as part of a healthy lifestyle) should be updated taking optimal levels into account.

## The longevity revolution within a context of increasing inequalities

The world is ageing rapidly. A baby born in 2015, can expect to live to be 100—a trajectory that has been to this time a much rarer event. Life expectancy in a country such as Brazil was only 43 years in 1945—today, it is 77 years. This extraordinary achievement—termed, the longevity revolution (LR)—will result in a tenfold increase in the worldwide 60 + population between 1950 and 2050. By 2050, the 80 + age group will have increased 27 times. In the same time period, the total population will only have increased 3.7 times.

But it is not only the speed of the LR that is so remarkable and challenging. It is also the context to that ageing. Developed countries first experienced wealth, and then became aged. In contrast, developing countries are ageing much faster and within a context of persistent levels of hardship. For example, in France the percentage of 60 + has doubled since 1850, while in Brazil, Thailand, and China the same doubling will take a mere 20 years (before 2030).

Social and economic inequalities are major contributors to the health disparities that have led to rapid (or premature) ageing in many developing countries. In these countries, premature ageing is characterised by high rates of preventable (non-communicable) disease, injury, and social exclusion. This has implications at both the individual and population levels, as both intrinsic capacity (a composite of all the physical and mental attributes on which an individual can draw—WHO) and functional ability (the health-related attributes that enable people to be and to do what they have reason to value—WHO) are adversely affected.

But ageing and health disparities can affect even wealthy developed countries. For example, the US boasts the largest economy in the world but ranks 32 on life expectancy at birth (LEB) due to drastic social and health inequalities. In fact, the US LEB is even on the decline, and has shown signs of reversing secular trends.

Examples of policies aimed at providing better standards include New Zealand, which recently announced a commitment to national wellbeing, and South Australia, which created a Centre for Well-Being and Resilience back in 2013. Despite these strides, the “modern plagues”—over nutrition, obesity, non-communicable diseases (NCD’s)—remain widespread, and all of which could be mitigated or prevented through appropriate policies that promote a healthy diet.

Policies to support individuals to age healthfully, include, but go beyond appropriate nutrition and health policies. Appropriate policies are needed throughout the life course. *The earlier the better but it is never too late* is a mantra that everyone should embrace.

**Fig. 3:**
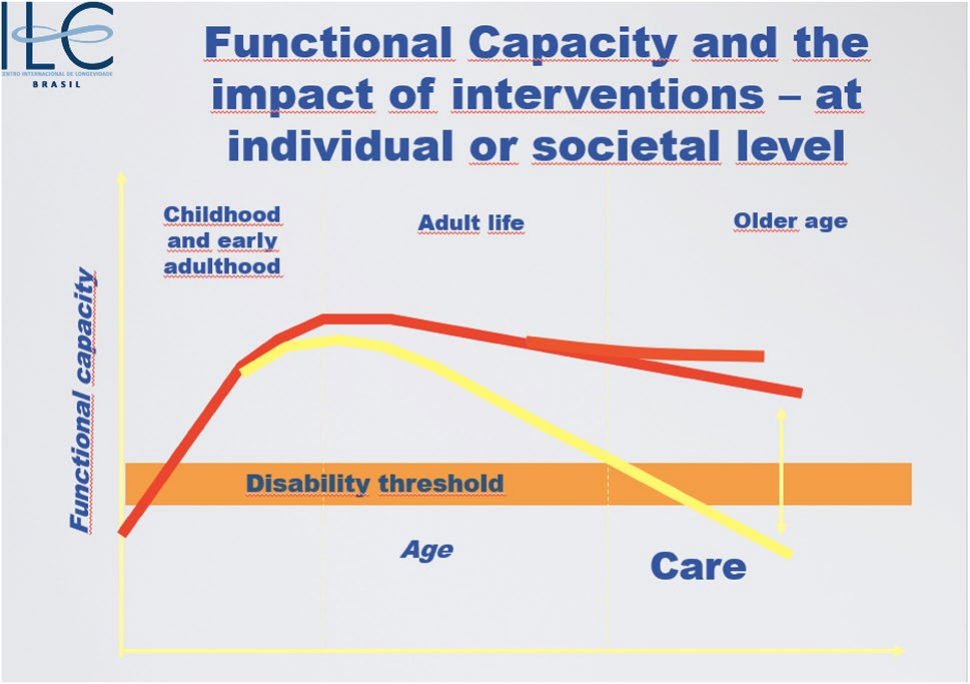
Interventions (such as nutrition) and robust public policy are needed to positively affect the functional capacity trajectory as we age (internal ILC figure)

In fact, this mantra applies to the four “capitals” that we need to accumulate as we age: *vital* (health); *knowledge* (life-long learning); *social* (participation) and *financial* capital (security). Individuals must bear a lot of the responsibility to make appropriate adjustments to their life trajectories but without robust public policies *to make the good choices*, *the easy and more affordable choices,* their efforts will be constantly undermined. If and when that is done, we will be on the road to developing a genuine *culture of care* that is vital for our fast ageing populations and is commensurate with a truly modern society (see Fig. 3).

## Conclusion

Global population data demonstrate that humans are indeed living longer, with many countries and population subsets living 30 years longer than just a century ago. However, this increase in lifespan has not always been paralleled by improvements in healthspan. Failure to recognize the differing nutritional and health needs of the aged, and burdened by social and economic inequalities, a rapidly ageing population has also experienced increased rates of non-communicable disease and healthcare costs. Healthspan rather than lifespan, needs to be recognized as the goal for all stages of a society, optimizing the quality and capabilities such that our final years are not faced with unnecessary deterioration and trepidation, but anticipation as active contributors to society. A combination of policies aimed at reducing social inequalities with innovative nutritional approaches have the potential to delay premature ageing and improve healthspan.

The World Health Organization is proposing the “Decade of Healthy Ageing 2020–2030” [71] to “explore synergies between policy instruments to catalyze meaningful and measurable impact to improve older peoples’ lives”. The reader is encouraged to visit WHO’s website, read the call-to-action spelled out in the 2015 WHO Global Strategy and Action Plan on Ageing and Health 2016–2030 and engage in the conversation [70]. This strategic approach, including appropriate, science-based nutritional interventions, should be developed and adapted for use in all countries independent of their level of economic development to support healthy ageing throughout the lifespan.
